# Leakage Performance of the GM + CCL Liner System for the MSW Landfill

**DOI:** 10.1155/2014/251465

**Published:** 2014-02-25

**Authors:** Fan Jingjing

**Affiliations:** Changsha Environmental Protection Vocational College, Changsha 410004, China

## Abstract

The contaminants in the landfill leachate press pose a grave threat to environment of the soil and the groundwater beneath the landfill. Despite there being strict requirements in relevant provisions of both domestic and foreign countries for the design of the bottom liner system. Pollution of the soil and the groundwater still took place in a number of landfills because of the leakage. To investigate the leakage rate of the liner systems, the minimum design requirements of the liner systems are summarized according to the provisions of four countries, including China, USA, Germany, and Japan. Comparative analyses using one-dimensional transport model are conducted to study the leakage performance of these liner systems composed of geomembrance (GM) and compacted clay layer (CCL) meeting the relevant minimum design requirements. Then parametric analyses are conducted to study the effects of the hydraulic head, the thickness of GM, the hydraulic conductivity of CCL, and so forth on the leakage performance of the liner system. It is concluded that the liner system designed according to the minimum design requirements of Germany provide the best antileakage performance, while that of Japan performs the lowest. The key parameters affecting the failure time of the liner system are summarized. Finally, some suggestions for the design of the liner systems are made according to the analyses.

## 1. Introduction

With the rapid urbanization in the developing countries [1–3], the increased municipal waste resulted in serious problems of the pollution [4–7]. To eliminate the adverse effects of the municipal waste to the environment, we make use of composting, incineration, and landfill to dispose the waste [8–10]. Within these approaches, the landfill is the most widely used one because it has the advantage of simplified technique, large capacity, and cost saving [11–13]. However, the leachate produced by the municipal wastes in the landfill pressed serious threat of pollution to the soil and the groundwater beneath the landfill because of the high concentration of toxic pollutants in the leachate [14–17].

To prevent the pollution of the soil and the groundwater from the leachate, we employ the liner system to block the leachate from the surroundings. The most widely used liner system is composed of a high-density polyethylene (HDPE) geomembrance (GM) over a compact clay layer (CCL) [18–23]. A number of countries, including China [24], US [25], Germany [26], and Japan [27], have released technique specifications on the liner system based on their independent experimental and analysis research. Rowe and Booker [28] conducted comparative research on three kinds of the liner system, including GM + CCL, GM + CCL + attenuation layer (AL), and the double lined system. Rowe concluded that the standard barrier designs eventually gave rise to unacceptable impacts due to the clogging effects of the leachate collection system. Xie et al. [29] studied four types of landfill liner systems which were proposed in the Chinese specification. The conclusion was that the double-layered GM + CCL liner system was the best barrier for the contaminant. Turan et al. [30, 31] conducted an intensive study on the performance of the different liner materials with bentonite on the removal efficiency of Cu(II) and Zn(II) from the leachate. Some design recommendations were made to improve the removal efficiencies of the liner system.

Previous studies mainly paid their attention to the leakage rate of the liner systems with different types of structures. For the GM + CCL liner system, which is the most widely used liner system, the minimum requirements are different from one specification to another. The performance of the GM + CCL liner system satisfying the minimum requirements of different specifications is rarely studied. To investigate the barrier performance of the GM + CCL liner systems that meet the minimum design requirements of different specifications, we conduct a comparative study on four kinds of GM + CCL liner systems proposed in the specification of China, USA, Germany, and Japan in terms of leakage rate, mass concentration, and contaminant critical time. Then parametric analyses are conducted to study the influence of water head, thickness of GM, thickness of CCL, and so forth on the leakage performance of the liner system. Some propositions for the corresponding design provisions are proposed according to the parametric analyses.

## 2. Minimum Design Requirements for the Liner System

**Figure 1:**
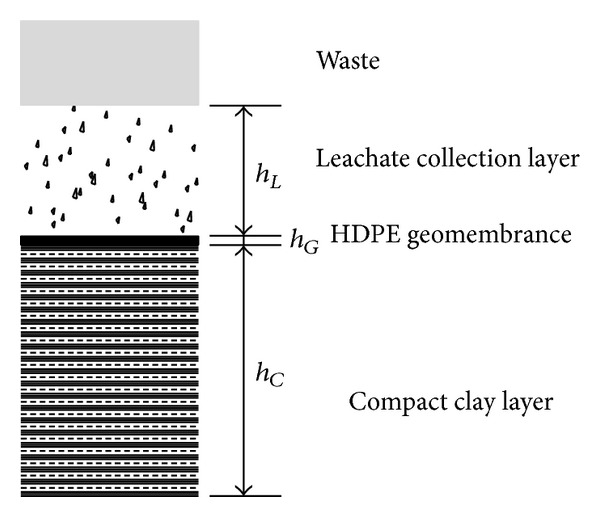
Schematics of the bottom liner system for MSW.

The schematics of the GM + CCL bottom liner system for MSW are shown in Figure 1. The function of the leachate collection layer is to collect and discharge the leachate produced by the waste. *h*_*L*_ is the hydraulic head of the leachate collection layer. The high-density polyethylene (HDPE) geomembrance (GM) and the compact clay layer (CCL) are placed behind the leachate collection layer to prevent the contaminant from invading the subsoil and groundwater. *h*_*G*_ and *h*_*C*_ are the thickness of the HDPEGM and CCL, respectively.

The types of GM + CCL liner systems examined are as follows.

- Chinese specification—CJJ113-2007 Technical code for liner system of municipal solid waste landfill [24] proposed four types of liner systems, including GM + CCL, GM + geosynthetic clay liner (GCL), a single CCL, and a single GM. The GM + CCL liner system is composed of a CCL with a thickness of 0.75 m covered by the HDPE GM with a thickness of 1.5 mm. The coefficient of hydraulic conductivity of the CCL should be less than 1 × 10^−9^ m/s.
- US subtitle D minimum design [25] consists of a 1.5 mm thick HDPEGM over a 0.6 m thick CCL with a hydraulic conductivity of 1 × 10^−9^ m/s.
- German minimum design requirements for the liner system are composed of a 2.5 mm thick HDPEGM over a 0.75 m thick CCL with a hydraulic conductivity of 5 × 10^−10^ m/s [26].
- The Japanese guideline [27] specifies minimum design requirements for the liner system consisting of a 1 mm thick HDPEGM and a 0.5 m thick CCL with a hydraulic conductivity of 1 × 10^−8^ m/s.

The minimum requirements for the liner system are summarized in Table 1. *k*_*c*_ is the maximum hydraulic conductivity of the CCL. The meaning of the other items is shown in Figure 1.

## 3. One-Dimensional Finite Layer Model

### 3.1. Theory

The main mechanisms of the contaminant transporting through the liner system are advection and diffusion (including mechanical dispersion). One-dimensional (1D) contaminant transport analysis is performed to study the performance of the liner system. The assumptions are as follows [32, 33].

*(a) The Source of the Contaminant Is on the Top of the Liner System.* The flow in each layer during each time interval is steady state. The transient flow conditions are not considered. This assumption is checked by Rowe and Nadarajah [34]. They concluded that the neglect of the transient flow condition has little influence on the engineering practice. 

*(b) The Properties of Each Layer Are Uniform.* These properties include the diffusion coefficient, porosity, and advective velocity. Different layers may have different properties and these properties may change at different times.

*(c) The Sorption Is First Order*. Based on the assumption above, the advective-diffusive-dispersive transport of the contaminant through the liner system is governed by the following:

(1) $$\begin{array}{c} R_{d}\frac{\partial C}{\partial t}=D\frac{\partial^{2}C}{\partial z^{2}}-v\frac{\partial C}{\partial z}-\lambda C, \end{array}$$

where *C* is the concentration of the contaminant, *D* is the coefficient of the hydrodynamic dispersion in the layer, *v* is the Darcy velocity, and *λ* is the first order sink term for modeling the contaminant removed per unit volumn:

(2) $$\begin{array}{c} \lambda =\frac{\ln2}{T_{1/2}}\;, \end{array}$$

where *T*_1/2_ is the half-life of the contaminant, and *R*_*d*_ is the decay coefficient:

(3) $$\begin{array}{c} R_{d}=1+\frac{\rho K_{d}}{n}, \end{array}$$

where *ρ* is the dry density of the layer, *K*_*d*_ is the distribution coefficient, and *n* is the porosity of the layer.

The finite layer technique proposed by Rowe and Booker [32] is adopted to solve the governing equation. The solving steps include

1. simplifying the partial differential equation by performing a Laplace transform and solving the simplified equation analytically;
2. performing an inverting Laplace transform to the solution in the previous step numerically and obtaining the numerical solution of the governing equation.

### 3.2. Landfill Model

The landfill considered has a length of 200 m in the direction of the groundwater flow. Two contaminants are considered. They are Cd^2+^ and the dichloromethane. The two contaminants are chosen because they are common in the leachate and the data of concentration are easily available. Cd^2+^ represents the heavy metal in the leachate. The initial concentration for Cd^2+^ is 1 mg/L [29]. The dichloromethane represents the organic pollutant in the leachate. The initial concentration of the dichloromethane is 5 mg/L [35]. According the US drinking water standards, the maximum contaminant level (MCL) for Cd^2+^ and the dichloromethane are 0.01 mg/L and 0.005 mg/L, respectively. The relative critical concentration for Cd^2+^ and the dichloromethane are 0.01 and 0.001, respectively. The hydraulic conductivity, porosity, and dry density are related to each other. The relationship proposed by Mesri and Braja [36, 37] is adopted, as listed in Table 2. The contaminant characteristics are determined according to Rowe et al. [38], as shown in Table 3.

## 4. Analysis Results and Discussion

**Figure 2:**
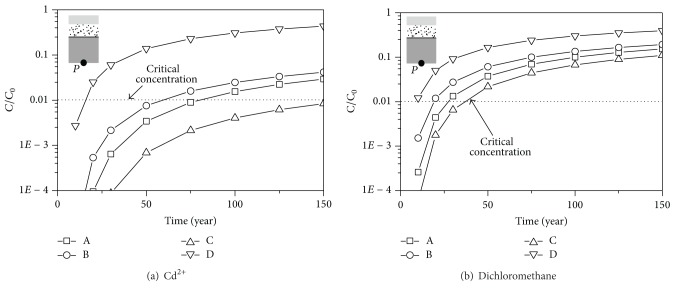
The concentration-time curves of the contaminants.

The concentration versus time curves is shown in Figure 2. The concentration increases with the increase of the time. For a given time, the concentration of the contaminant is the highest for the liner system of the Japanese standard, followed by that of the US standard and that of the Chinese standard. The concentration of the contaminant is the lowest for liner system of the German standard. The reason is that the thickness of the GM and the CCL are the largest and the hydraulic conductivity is the smallest in the liner system of the German standard. While the thicknesses of the GM and the CCL are the smallest and the hydraulic conductivity is the largest in the liner system of the Japanese standard, it is indicated that the minimum requirements in German standard are the most conservative, while those in Japanese standard are the most economic.

The time at which the concentration at the bottom of the liner system reaches the MCL is defined as the critical time. The critical times of the four types of the liner system are listed in Table 4. The liner system of the German standard has the longest critical time, while that of the Japanese standard has the shortest critical time. The critical time of the dichloromethane is shorter than that of Cd^2+^ because the diffusion coefficient of the dichloromethane is larger than that of Cd^2+^.

**Figure 3:**
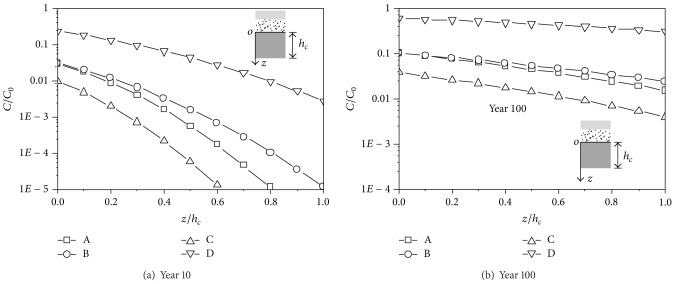
The concentration-depth curves of the Cd^2+^.

**Figure 4:**
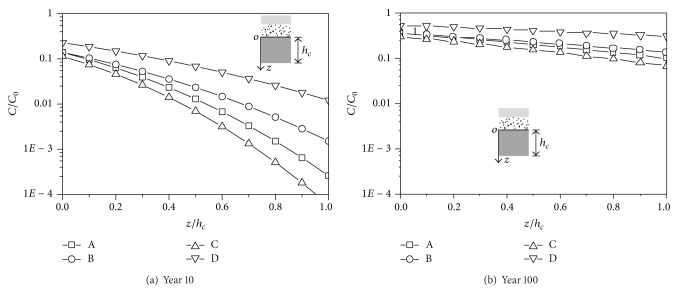
The concentration-depth curves of the dichloromethane.

The concentration versus depth curves is shown in Figures 3 and 4. At year 10, the concentration at the top of CCL (*z* = 0) is the largest for the Japanese liner system and is the smallest for the German liner system. The concentration at the top of CCL is very close for the US liner system and the Chinese liner system. At year 100, the distribution of the concentration at the top of CCL is similar to that at year 10. The gradient of the concentration at year 100 is smaller than that at year 10, which indicates that the concentration along the depth tends to be uniformly distributed with the increase of time. The difference of the concentrations at the top of CCL for Cd^2+^ is greater than that for the dichloromethane. The reason might be that the difference of the diffusion coefficient in GM and CCL for Cd^2+^ is greater than that for dichloromethane.

## 5. Parametric Analysis

To investigate the performance of the GM + CCL liner system comprehensively, parametric analyses are conducted based on the Chinese liner system. The values of the parameters studied are listed in Table 5. The parameters include the hydraulic head of the leachate collection system *h*_*w*_, the thickness of GM *h*_*G*_, the defects of GM, the thickness of CCL *h*_*C*_, and the coefficient of conductivity. Because of time and space limitations, attention is paid to the migration behavior of Cd^2+^.

### 5.1. Hydraulic Head of the Leachate Collection System

**Figure 5:**
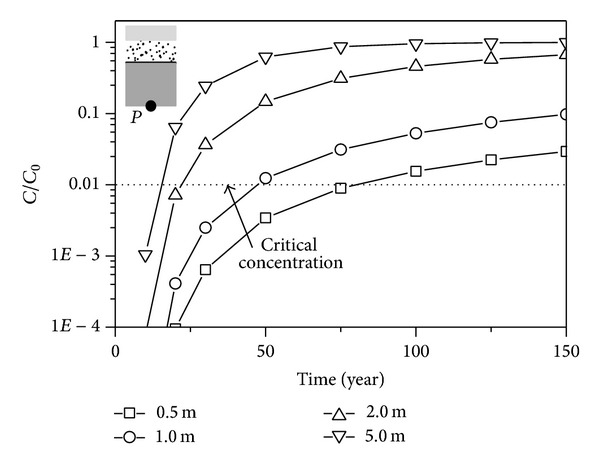
Comparison of numerical results with different water heads.

The function of the leachate collection system is to limit the head acting on the liner system. In the life-cycle of the landfill, the permeability of the leachate collection system decreases with the physical, chemical, and biological corrosion. If the hydraulic head of leachate collection system exceeds the design value, the leachate collection system is not able to perform its primary function and the liner system is easier to be broken through. This condition is called clogged. Four types of the hydraulic head are considered. They are 0.3 m, 1 m, 5.0 m, and 10.0 m. The concentration versus time curves is shown in Figure 5. The concentration increases with the increase of the hydraulic head. The critical times of the liner system with a hydraulic head of 0.3 m, 1 m, 5.0 m, and 10.0 m are 79.1 years, 45.3 years, 21.0 years, and 11.4 years, respectively.

### 5.2. The Thickness of the GM

**Figure 6:**
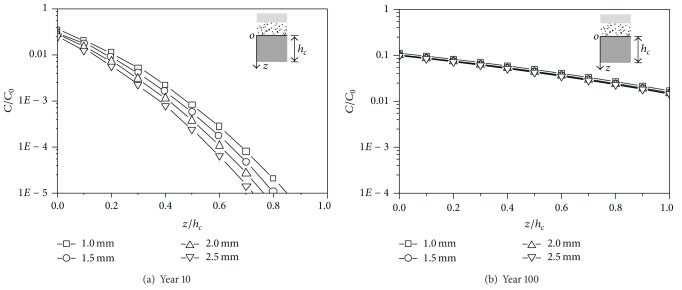
Comparison of numerical results with different GM thicknesses.

The seepage performance of the GM has been widely recognized with the development of the geotextile material technology. The GM has good durability and elongation properties subjected to tension. It is economic in transportation and construction. Therefore, the GM is widely used in the landfill liner system. The thickness of the GM studied includes 1 mm, 1.5 mm, 2 mm, and 2.5 mm. The concentration versus depth curves for different thickness is shown in Figure 6. The concentration of the contaminant at the top of CCL is insensitive to the thickness of the GM. The influence of the thickness of the GM to the seepage behavior of the liner system decreases with the increase of time. At year 100, the concentrations of the contaminant are almost equal for different GM thicknesses.

### 5.3. The Defects of GM

**Figure 7:**
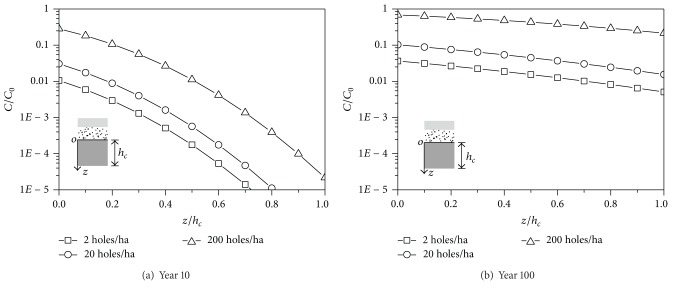
Comparison of numerical results with different GM defect holes.

The GM is prone to puncturing in the production and construction process, which results in defects of GM. The leakage of the contaminant through the defects is believed to be the main migration way through the GM [39]. Well, ordinarily, and badly constructed GM are assumed to have a defect number of 2 holes/ha, 20 holes/ha, and 200 holes/ha, respectively. The concentration versus depth curves for different numbers of defects is shown in Figure 7. The concentration at the top of CCL increases significantly with the increase of defects. The influence remains with the increase of the time, which confirms that the migration of the pollutants into the GM is mainly caused by the leakage through the defects, rather than by diffusion.

### 5.4. The Thickness of the CCL

**Figure 8:**
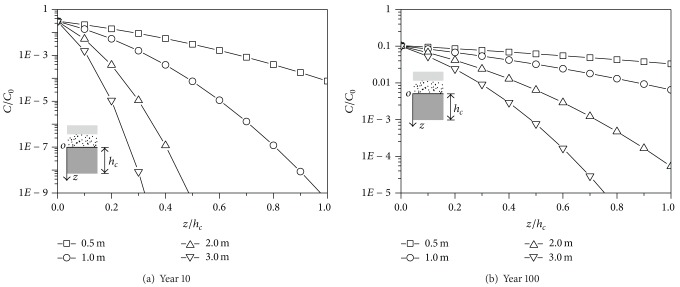
Comparison of numerical results with different CCL thicknesses.

**Figure 9:**
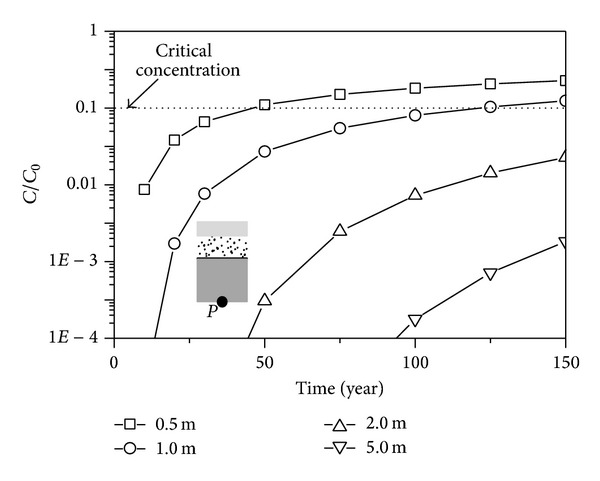
Comparison of concentration-time with different CCL thicknesses.

The concentration versus depth curves for various thicknesses of CCL is shown in Figure 8. The concentration of the contaminant at the top of CCL remains with the increase of the thickness of CCL. It is indicated that the thickness of the CCL has little effect on the concentration at the top of CCL. The concentration at the bottom of CCL decreases remarkably with the increase of the thickness of CCL. The concentration at the bottom of CCL versus time curves is shown in Figure 9. The critical times increase almost three times when the thickness of the CCL increases from 0.5 m to 1.0 m, which indicates that increasing the thickness of the CCL is beneficial to the seepage behavior of the liner system.

### 5.5. The Hydraulic Conductivity of the CCL

**Figure 10:**
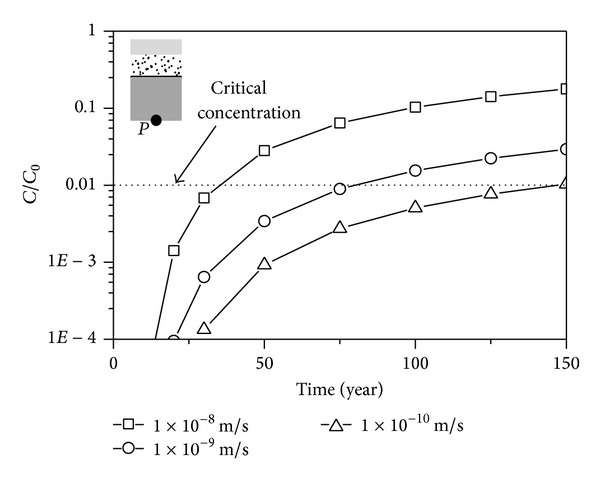
Comparison of numerical results with different conductivities.

The minimum design requirements of hydraulic conductivity are different from one country to another. The effects of the hydraulic conductivity on the seepage behavior of the liner system are shown in Figure 10. The concentration decreased notably with the decrease of the hydraulic conductivity. The critical times of the liner system are 33 years, 79.1 years, and 146.7 years when the values of the hydraulic conductivity are 1 × 10^−8^ m/s, 1 × 10^−9^ m/s, and 1 × 10^−10^ m/s, respectively, which indicates that the seepage behavior of the liner system improve significantly with the decrease of the hydraulic conductivity.

### 5.6. Leakage Characteristics of the Liner Systems

The concentration at the top of CCL, the concentration at the bottom of CCL, and the critical time are selected as the leakage characteristics of the liner systems. The leakage characteristics are listed in Table 6. The parameters affecting the concentration at the top of CCL include the hydraulic head, defects of the GM, and the hydraulic conductivity of the CCL. The concentration at the top of CCL increases with the increase of hydraulic head, number of defects on the GM, and the hydraulic conductivity of the CCL. The influence of the hydraulic head increases with the increase of time, while that of the number of the defects and the hydraulic conductivity are insensitive with the changing of time. The main factors influencing the concentration at the bottom of CCL are the hydraulic head, the number of the defects, the thickness of the CCL, and the hydraulic conductivity. The concentration at the bottom of the CCL increases with the increase of the hydraulic head, the number of defects, and the hydraulic conductivity, while the concentration at the bottom of the CCL decreases with the increase of the thickness of the CCL. The influences of the hydraulic head and the number of defects on GM to the bottom concentration are insensitive to the increase of time. The influences of the thickness of CCL and the hydraulic conductivity to the bottom concentration increase rapidly with the increase of time. The key factors affecting the critical time are the hydraulic head, the number of defects, the thickness of CCL, and the hydraulic conductivity. The critical time decreases with the increase of the hydraulic head, the number of defects on GM, and the hydraulic conductivity. The critical time increases with the increase of the thickness of the CCL. Therefore, the ways to improve the leakage behavior of the liner system include reducing the hydraulic head of the leachate collection system and the hydraulic conductivity of the CCL, controlling the quality of the construction to lessen the number of defects on the GM and increasing the thickness of the CCL.

## 6. Conclusion

The finite layer model is adopted to simulate the leakage behavior of the landfill liner system. The minimum design requirements of four countries for the liner system are compared. Parametric analyses are conducted to study the main parameter affecting the leakage behavior of the liner system. The conclusions are as follows.

1. The minimum designed liner system according to the German specification has the best antileakage behavior, while that according to the Japanese specification has the worst antileakage performance among the minimum designed liner systems in China, US, German, and Japan. The performance of the minimum designed liner systems in China and US is similar and locates in the middle of that among the four counties.
2. The thickness of the GM has little effects on the critical time, the concentration at the top of the CCL, and the concentration at the bottom of the CCL. The main way for the migration of the contaminant in the GM is through the defects.
3. The concentration at the top of CCL increases with the increase of the hydraulic head, the number of defects on GM, and the hydraulic conductivity. The bottom concentration becomes larger with the increase of the hydraulic head, the number of defects on GM, and the hydraulic conductivity. The bottom concentration decreases with the increase of the thickness of the CCL.
4. The critical time of the liner system lessens with the increase of hydraulic head, the number of defects on GM, and the hydraulic conductivity, while the critical time increases with the increase of the thickness of the CCL. The ways to control the leakage behavior of the liner system are reducing the hydraulic head, the number of defects on GM, and the hydraulic conductivity, as well as increasing the thickness of the CCL.

## Figures and Tables

**Table 1:** Minimum requirements for the liner system.

Item	China	USA	Germany	Japan
*h*_*L*_ (m)	0.3	0.3	0.3	0.5
*h*_*G*_ (mm)	1.5	1.5	2	1
*h*_*C*_ (m)	0.75	0.6	0.75	0.5
*k*_*C*_ (m^2^/s)	1 × 10^−9^	1 × 10^−9^	1 × 10^−10^	1 × 10^−8^

**Table 2:** Hydraulic conductivity, porosity, and dry density in CCL.

Property	Coefficient of conductivity *k*_*C*_ (m^2^/s)		
	1 × 10^−8^	1 × 10^−9^	1 × 10^−10^
Porosity *n*	0.42	0.35	0.28
Dry density (g/cm^3^)	1.69	1.79	1.90

**Table 3:** Characteristics of the contaminant transportation.

Item	Cd^2+^	Dichloromethane
Initial concentration (mg/L)	1	5
MCL (mg/L)	0.01	0.005
Diffusion coefficient of GM (m^2^/s)	6 × 10^−15^	2.1 × 10^−13^
Diffusion coefficient of CCL (m^2^/s)	1.76 × 10^−10^	5.7 × 10^−10^
Adsorption coefficient of CCL *K*_*d*_ (mL/g)	0.36	0.8

**Table 4:** Critical times of the liners/year.

Type	Cd^2+^	Dichloromethane
China	79.1	20.7
USA	57.3	13.4
Germany	168.4	26.8
Japan	13.3	8.1

**Table 5:** Variables for parametric study.

Parameters	Value			
Hydraulic head/m	0.3	1.0	5.0	10.0
The thickness of GM/mm	1.0	1.5	2.0	2.5
The defects of GM/holes/ha	2	20	200	—
The thickness of CCL/m	0.5	1.0	2.0	3.0
Coefficient of conductivity/ms^−1^	1 × 10^−8^	1 × 10^−9^	1 × 10^−10^	—

**Table 6:** Leakage characteristics of the liner systems.

Parameters	Value	Top concentration *C*/*C*_0_		Bottom concentration *C*/*C*_0_		Critical time (year)
		Year 10	Year 100	Year 10	Year 100	
Hydraulic head (m)	0.3	3.06 × 10^−2^	4.11 × 10^−7^	1.03 × 10^−1^	1.55 × 10^−2^	79.1
	1.0	9.51 × 10^−2^	2.52 × 10^−6^	2.87 × 10^−1^	5.28 × 10^−2^	45.3
	5.0	4.66 × 10^−1^	7.81 × 10^−5^	8.99 × 10^−1^	4.59 × 10^−1^	21.0
	10.0	8.13 × 10^−1^	1.05 × 10^−3^	9.99 × 10^−1^	9.58 × 10^−1^	11.4

The thickness of GM (mm)	1.0	3.50 × 10^−2^	1.11 × 10^−1^	8.85 × 10^−7^	1.70 × 10^−2^	75.3
	1.5	3.06 × 10^−2^	1.03 × 10^−1^	4.11 × 10^−7^	1.55 × 10^−2^	79.1
	2.0	2.77 × 10^−2^	9.95 × 10^−2^	1.81 × 10^−7^	1.48 × 10^−2^	81.1
	2.5	2.48 × 10^−2^	9.81 × 10^−2^	7.55 × 10^−8^	1.44 × 10^−2^	82.6

The defects of GM (holes/ha)	2	1.06 × 10^−2^	3.65 × 10^−2^	1.15 × 10^−7^	5.09 × 10^−3^	153.0
	20	3.06 × 10^−2^	1.03 × 10^−1^	4.11 × 10^−7^	1.55 × 10^−2^	79.1
	200	2.84 × 10^−1^	6.86 × 10^−1^	2.17 × 10^−5^	2.15 × 10^−1^	27.3

The thickness of CCL (m)	0.5	3.11 × 10^−2^	1.04 × 10^−1^	7.57 × 10^−5^	3.30 × 10^−2^	44.3
	1.0	3.04 × 10^−2^	1.02 × 10^−1^	4.79 × 10^−10^	6.47 × 10^−3^	120.4
	2.0	3.00 × 10^−2^	1.01 × 10^−1^	3.91 × 10^−18^	5.31 × 10^−5^	905.2
	5.0	2.99 × 10^−2^	1.00 × 10^−1^	9.88 × 10^−26^	4.37 × 10^−8^	>1000

The coefficient of conductivity (m/s)	1 × 10^−8^	1.48 × 10^−1^	4.18 × 10^−1^	1.90 × 10^−5^	1.03 × 10^−1^	33.0
	1 × 10^−9^	3.06 × 10^−2^	1.03 × 10^−1^	4.11 × 10^−7^	1.55 × 10^−2^	79.1
	1 × 10^−10^	1.35 × 10^−2^	4.66 × 10^−2^	2.25 × 10^−8^	5.08 × 10^−3^	146.7
